# DruGagent: Multi-Agent Large Language Model-Based Reasoning for Drug-Target Interaction Prediction

**Published:** 2025-04-07

**Authors:** Yoshitaka Inoue, Tianci Song, Xinling Wang, Augustin Luna, Tianfan Fu

**Affiliations:** Dept of Computer Science and Engineering, University of Minnesota, Minneapolis, MN, USA; Computational Biology Branch, National Library of Medicine, Developmental Therapeutics Branch, National Cancer Institute, Bethesda, MD, USA; Dept of Computer Science and Engineering, University of Minnesota Minneapolis, MN, USA; Khoury College of Computer Sciences, Northeastern University Arlington, VA, USA; Computational Biology Branch, National Library of Medicine, Developmental Therapeutics Branch, National Cancer Institute, Bethesda, MD, USA; Department of Computer Science, Nanjing University, Nanjing, Jiangsu, China

## Abstract

Advancements in large language models (LLMs) allow them to address diverse questions using human-like interfaces. Still, limitations in their training prevent them from answering accurately in scenarios that could benefit from multiple perspectives. Multi-agent systems allow the resolution of questions to enhance result consistency and reliability. While drug-target interaction (DTI) prediction is important for drug discovery, existing approaches face challenges due to complex biological systems and the lack of interpretability needed for clinical applications. DrugAgent is a multi-agent LLM system for DTI prediction that combines multiple specialized perspectives with transparent reasoning. Our system adapts and extends existing multi-agent frameworks by (1) applying coordinator-based architecture to the DTI domain, (2) integrating domain-specific data sources, including ML predictions, knowledge graphs, and literature evidence, and (3) incorporating Chain-of-Thought (CoT) and ReAct (Reason+Act) frameworks for transparent DTI reasoning.

We conducted comprehensive experiments using a kinase inhibitor dataset, where our multi-agent LLM method outperformed the non-reasoning multi-agent model (GPT-4o mini) by 45% in F1 score (0.514 vs 0.355). Through ablation studies, we demonstrated the contributions of each agent, with the AI agent being the most impactful, followed by the KG agent and search agent. Most importantly, our approach provides detailed, human-interpretable reasoning for each prediction by combining evidence from multiple sources - a critical feature for biomedical applications where understanding the rationale behind predictions is essential for clinical decision-making and regulatory compliance. Code is available at https://anonymous.4open.science/r/DrugAgent-B2EA.

## 1 Introduction

Large Language Models (LLMs) have demonstrated remarkable capabilities in solving a wide range of problems using human-friendly inputs (Wei et al., 2022a). However, these models still face limitations when confronted with tasks outside their training scope or those requiring real-time data access and specialized domain knowledge. To address these challenges, there is a growing interest in Multi-Agent systems (Du et al., 2023) that utilize several agents to talk to each other and make a final decision incorporating external tools such as Knowledge Graphs (KG) (Shu et al., 2024), and Retrieval-Augmented Generation (RAG) (Lewis et al., 2020). These systems offer a more robust and reliable approach to problem-solving by leveraging diverse information sources and specialized capabilities. In this paper, we propose a multi-agent system that integrates unstructured text, structured knowledge graphs, and machine learning predictions.

Our research applies this multi-agent approach to the challenge of drug-target interaction (DTI) prediction, a strategy that can reduce the time, cost, and risk associated with drug development (Abbasi Mesrabadi et al., 2023).

Pharmaceutical research faces high failure rates due to the complexity of biological systems and the diversity of biomedical information sources (Chen et al., 2024b; Wu et al., 2022b). While recent advances in artificial intelligence have helped address these challenges (Vamathevan et al., 2019), the effective integration of heterogeneous data sources remains a major research area.

We propose a multi-agent system architecture where each agent specializes in a specific aspect of the drug discovery process. Our architecture includes five agents: Coordinator, AI, KG, Search, and Reasoning Agent. The Coordinator Agent manages communication and data formatting between agents. The AI Agent employs the DeepPurpose package to predict DTI scores from molecular structures and protein sequences. The KG Agent calculates path-based interaction scores from biomedical knowledge graphs. The Search Agent analyzes search results to extract evidence and score interactions through keyword matching. Each specialist agent provides both scores and reasoning for their outputs - the AI Agent explains its ML-based prediction, the KG Agent describes discovered path relationships (e.g., ‘3-hop connection via LYN’), and the Search Agent provides literature-based evidence. The Reasoning Agent then integrates this evidence through CoT and ReAct frameworks to generate final scores with step-by-step explanations.

The architecture we developed, although initially designed for biological applications, can be adapted to various other fields requiring multiple perspectives.

## 2 Related Works

### Machine Learning in Drug Target Interaction

Machine learning (ML) techniques have aided drug discovery, with use in various aspects of pharmaceutical research. DeepPurpose (Huang et al., 2020) is a deep learning model for DTI prediction that combines several algorithms (i.e., Graph Neural Networks (GNNs) and Convolutional Neural Networks (CNNs)). This model offers a versatile pre-trained approach applicable to a wide range of drug discovery tasks such as DTIs and drug property predictions.

#### Knowledge Graphs for Integrative Analysis.

Knowledge graphs provide a structured way of integrating diverse biological data. For instance, the DRKG integrates data from several sources, including DrugBank (Knox et al., 2024), Hetionet (Himmelstein et al., 2017), and STRING (Szklarczyk et al., 2023), to offer comprehensive insights into possible drug-disease links (Ioannidis et al., 2020).

#### Literature Search using LLMs

The automation of literature review and data extraction using AI tools, particularly LLMs, has become a component of modern drug discovery (Chakraborty et al., 2023). Recent studies have demonstrated that LLM-based search tools can enhance the efficiency and complexity of queries compared to traditional search engines (Spatharioti et al., 2023).

#### LLMs with Reasoning

LLMs with reasoning is a current trend in 2024. With several techniques, such as the Chain-of-Thought (CoT) (Wei et al., 2022b) and ReAct (Reason+Act)(Yao et al., 2022), LLM can make a reason why it makes a decision. CoT enables LLMs to show their reasoning process by generating intermediate steps, such as “Let’s approach this step by step: First, analyze the ML prediction score..., Second, examine the knowledge graph evidence..., Finally, integrate all evidence...”. ReAct extends this by combining reasoning with actions, following a ”Thought → Action → Observation” pattern where the model first reasons about what to do, then takes an action (e.g., analyzing evidence), and observes the results to inform the next step. These reasoning capabilities enable LLMs to break down complex problems into smaller steps and provide more explainable outputs (Xu et al., 2025).

### Multi-Agent Systems

Multi-agent systems (MAS) have evolved significantly since their inception. Early work by Smith (1980) introduced the Contract Net Protocol for distributed problem solving, while Wooldridge & Jennings (1995) established fundamental agent architectures and interaction protocols. Bellifemine et al. (2005) developed JADE, a framework that standardized agent development. Recently, LLM-based autonomous agents have gained tremendous interest in several topics, such as medicine and finance (Samvelyan et al., 2019; Chen et al., 2024a; Tang et al., 2023; Akata et al., 2023).

## 3 Methods

### 3.1 Overview of Drugagent

Our proposed system is a conversational multi-agent architecture analogous to a specialized research team focused on drug-target interaction prediction. Each agent plays a distinct role, mirroring separate tasks research team members would do: some focus on ML models, others on search-based analysis, and another is dedicated to knowledge graph exploration and making final decisions.

The system employs LLMs for natural language processing and response generation, enhances reasoning through step-by-step problem-solving methodologies, and performs actions like calculating scores, analyzing literature, and querying knowledge graphs. It then integrates this information using a weighted average approach to simulate a knowledgeable DTI research team.

The workflow of our system follows a systematic multi-agent approach:

1. The workflow begins with user input specifying drug and target names, initializing five specialized agents (Coordinator, AI, Search, KG, and Reasoning Agents).
2. The Coordinator Agent orchestrates the process by distributing tasks to agents and managing inter-agent communication through the AutoGen (Wu et al., 2023) GroupChat framework.
3. Each specialist agent executes tasks independently:
  - An AI Agent uses ML to predict DTI scores from molecular structures and protein sequences.
  - A Search Agent analyzes search results (title, link, content) to extract evidence of interactions.
  - A KG Agent calculates path-based interaction scores from biomedical knowledge graphs.
  - A Reasoning Agent integrates evidence from agents to generate a final score with a step-by-step explanation.
4. The system returns results in a CSV (comma-separated values) format containing scores from AI, KG, and Search Agent and final predictions with detailed explanations.

All numerical scores are normalized between 0 and 1, ensuring a consistent scale across different prediction methods.

Each agent utilizes different LLMs optimized for their specific tasks. The Coordinator Agent employs GPT-4o for complex task management and coordination, while AI, KG, and Search Agents use the lighter GPT-4o mini model. The AI Agent processes pre-trained model predictions, the KG Agent handles knowledge graph queries, and the Search Agent extracts information from search results. These specialized agents require less complex reasoning capabilities compared to the Coordinator Agent’s role in managing overall workflow and inter-agent communication. The Reasoning Agent, which analyzes and integrates evidence through CoT and ReAct frameworks, utilizes OpenAI o3-mini. This model was specifically fine-tuned for reasoning tasks, enabling it to evaluate evidence from multiple sources, assess their consistency, and generate final reasoning that combines insights from AI, KG, and Search Agents.

### 3.2 Agent Roles and Responsibilities

DrugAgent integrates several specialized agents. Through the use of advanced search capabilities, access to specialist models, and indexing in databases, these agents can execute a wide range of tasks. Below, we delve into the specific roles and responsibilities assigned to each agent within the system.

#### 3.2.1 Coordinator Agent

The Coordinator Agent manages the DTI prediction workflow by distributing specialized prompts to the AI, Knowledge Graph, Search, and Reasoning components. This agent is also responsible for input and output formatting through strictly defined prompt templates that specify the required output structure with Python List. For example, the AI Agent must return results in the format [drug name, target name, score], while the Reasoning Agent must provide [drug name, target name, AI Agent score, KG Agent score, Search Agent score, Final score from Reasoning Agent, Summarized reasoning by Reasoning Agent]. If any agent’s output deviates from these predefined formats, the system raises an error to maintain data consistency across the workflow.

#### 3.2.2 AI Agent

The AI Agent utilizes DeepPurpose (Huang et al., 2020) to predict potential drug targets, following the model format of the MPNN_CNN BindingDB model with binary drug response (sensitivity/resistance) prediction. This model combines Message Passing Neural Networks (MPNN) (Gilmer et al., 2017) for processing molecular structures with CNN for embedding binding site features. It is trained on the comprehensive BindingDB dataset, which contains binary binding affinity data for DTIs. This model can predict binding affinity values for any combination of SMILES and the target protein sequence provided. For evidence generation by the Reasoning Agent, the AI Agent provides a standardized reason for all predictions: “This agent used an ML model”.

#### 3.2.3 KG Agent

We construct a knowledge graph from multiple biomedical databases to enable comprehensive DTI analysis and provide interpretable reasoning for the discovered relationships in the graph.

##### Data Sources

The use of professional datasets is pivotal in ensuring the accuracy and reliability of our agents’ information retrieval capabilities.

- **DrugBank:** DrugBank (Knox et al., 2024) offers detailed drug data, including chemical, pharmacological, and pharmaceutical information, with a focus on DTIs. It provides data for over 13,000 drug entries, including FDA-approved small-molecule drugs, biopharmaceuticals (proteins, peptides, vaccines, and allergens), and nutraceuticals.
- **Comparative Toxicogenomics Database (CTD):** The CTD (Davis et al., 2023) is a curated database providing information about chemical–gene/protein interactions, chemical–disease, and gene-disease relationships (Chang et al., 2019; Wu et al., 2022a).
- **Search Tool for Interactions of Chemicals (STITCH):** STITCH (Kuhn et al., 2007) is a database of known and predicted interactions between chemicals and proteins. It integrates information from various sources, including experimental data and text mining of scientific literature.
- **Drug-Gene Interaction Database (DGIdb):** DGIdb (Cannon et al., 2024) is a resource that consolidates disparate data sources describing drug-gene interactions and gene druggability. It provides drug-target interaction and information on druggable genes used in cancer informatics, drug repurposing, and personalized medicine (Chen et al., 2021; Wang et al., 2024; Lu et al., 2024).

From these datasets, we create a unified drug-gene interaction table. This consolidated table contains 3,312 drugs and 23,066 genes. From this, we calculate the DTI score between the drug and target using the below formula:

(1) $$\text{DT}{\text{I}}_{\text{score}}(d,t)=\{\begin{array}{cc} 0 & \text{if}d\notin G\;\text{or}\;t\notin G\text{,}\\ \max_{p\in P(d,t)}\left\{\frac{w(p)}{\ln(1+|p|)}\right\} & \text{otherwise,} \end{array}$$

where d is a drug, t is a target, G is a knowledge graph, P(d,t) is the set of all paths between d and t up to length 4, and |p| is the number of hops in path p. We set the maximum path length to 4 as a proof of concept. The path weight w(p) is calculated as:

(2) $$w(p)=\frac{1}{\left|p\right|}\sum_{i=1}^{\left|p\right|}\frac{deg\left(n_{i}\right)+deg\left(n_{i+1}\right)}{2|V|},$$

where deg(n) represents the degree of node n (number of connections), and |V| is the total number of nodes in the graph. This weight calculation emphasizes paths through well-connected nodes, which are often more significant in biological networks. The final score is normalized to [0,1], where 1.0 indicates a direct interaction, and 0 indicates no valid path exists between the drug and target. The score decreases as path length increases or node importance decreases. For example, when calculating the relationship between Nilotinib and GRK5, our method finds multiple paths. Then, the highest-scoring path is selected, and this path information is utilized as a reason for final evidence by the Reasoning Agent.


Found 5 paths between Nilotinib and GRK5. Best score: 0.042
Path 1: Nilotinib → HSP90AA1 → Lauric acid → GRK5 (score: 0.042)
Path 2: Nilotinib → ABL1 → Dasatinib → GRK5 (score: 0.035)
Path 3: Nilotinib → MAPK14 → Imatinib → GRK5 (score: 0.028)

#### 3.2.4 Search Agent

Parallel to these processes, the Search Agent leverages LLMs to automate the extraction of relevant information from biomedical literature found via search engine hits. This agent summarizes a reason from search engine results (titles, links, and content.) by LLM and scores by using keyword matching for interaction terms. The search agent’s core functionality can be summarized as follows:

1. **Generate Search Query:** Construct a search query by combining the drug name, target name, and the term “interaction”
2. **Run Bing Search API:** Execute a Bing Search API query to retrieve titles, links, and content.
3. **Document Processing and Analysis:**
  - **Search Result Scoring:** Calculate interaction scores based on the presence of drug-target pairs, interaction keywords, and significance indicators in search results (Equations 3–7).
  - **Summary Generation:** Generate summaries of the search results using gpt-4o-mini.
4. **Evidence Integration:** Combine the calculated scores and generated summaries for the Reasoning Agent’s final integration.

The DTI score calculation is as follows: Let $R=r_{1},r_{2},\ldots ,r_{n}$ be the set of search results (titles, links, and content.), where n is the number of results (default n is 10). For each result $r_{i}$, we define an individual score function $S\left(r_{i}\right):S\left(r_{i}\right)=I\left(d,t,r_{i}\right)+I\left(p,r_{i}\right)+I\left(s,r_{i}\right)$, where

(3) $$I\left(d,t,r_{i}\right)=\{\begin{array}{cc} 1 & \text{if drug name}\;d\;\text{and target name}\;t\;\text{are in}\;r_{i}\\ 0 & \text{otherwise} \end{array}$$

(4) $$I\left(p,r_{i}\right)=\{\begin{array}{cc} 1 & \text{if any positive keyword is in}\;r_{i}\\ 0 & \text{otherwise} \end{array}$$

(5) $$I\left(s,r_{i}\right)=\{\begin{array}{cc} 1 & \text{if any strong keyword is in}\;r_{i}\\ 0 & \text{otherwise} \end{array}$$

The positive keywords are “interacts”, “binds”, “activates”, and “modulates”. The strong keywords are “strong”, “significant”, “potent”, and “effective”. The total score T is then calculated as:

(6) $$T=\sum_{i=1}^{n}S\left(r_{i}\right)$$

The maximum possible score M is M=3n. Finally, the normalized DTI score D is calculated as:

(7) $$D=\{\begin{array}{cc} \text{round}\left(\frac{T}{M},2\right) & \text{if}M>0\\ 0 & \text{if}M=0 \end{array}$$

where round (x,2) rounds x to 2 decimal places.

#### 3.2.5 Reasoning Agent

The Reasoning Agent integrates evidence from AI, KG, and Search Agent using CoT and ReAct frameworks. Through CoT, it breaks down complex evaluations into interpretable steps, while ReAct’s thought-action-observation pattern guides the systematic analysis process.

Shorten example output is as follows:


Thought: Analyze ML, KG, and Literature Search evidence for Vandetanib-
   MARK2 interaction.

Action: ANALYZE_EVIDENCE
Observation: ML = 7.26e-6, KG = 0.7213, Search = 0.3

Action: EVALUATE_MECHANISMS
Observation: KG shows 3-hop connection via LYN, literature supports
    kinase network interactions.

Action: CALCULATE_SCORES
Final Score = (0.00000726 + 0.72134752 + 0.3) / 3 = 0.34045

Final Output:
[“Vandetanib”, “MARK2”, 7.260064194269944e-06, 0.7213475204444817, 0.3,
    0.34045, “KG evidence (3-hop via LYN) and literature support moderate
    interaction despite near-zero ML score.”]


Full example output and prompts are in appendix A.1.

## 4 Experiment

### 4.1 Performance Comparison of Drugagent and Ablation Study

For quantitative evaluation, we used a kinase-compound activity dataset from a large-scale kinase profiling study (Anastassiadis et al., 2011). The dataset measures kinase activity as the percentage of remaining enzymatic function after compound exposure, normalized against solvent controls. We started with a dataset containing 300 protein kinases and 178 drugs (identified by CAS numbers). After converting the drug identifiers to SMILES and filtering proteins that present sequences in UniProt, our final dataset comprised 201 proteins and 75 drugs. For further evaluation, we generated five independent sets, each containing 50 kinase-drug combinations (250 combinations in total, containing 17 unique drugs and 149 unique proteins).

Table 1 shows the metrics for DrugAgent, non-reasoning model (GPT-4o mini) instead of the reasoning model (o3-mini) as baselines, and the ablation study including DrugAgent without Knowledge Graph Agent (w/o KG), without AI Agent (w/o AI), and Search Agent (w/o Search). We submitted 10 drug-target interactions at once following the ”Superposition” approach (Xiong et al., 2024), where multiple drug-target pairs are processed simultaneously rather than sequentially. For computational efficiency in ablation studies (w/o Search, w/o AI, w/o KG), we used the mean of the remaining components’ scores due to OpenAI API rate limits.

DrugAgent demonstrates a balanced prediction strategy in DTI analysis, achieving a precision of 0.571 compared to GPT-4o mini’s indiscriminate prediction approach (precision of 0.231). This selective prediction capability is particularly significant in drug discovery, where GPT-4o mini’s tendency to label all pairs as ”interacting” leads to perfect but misleading recall (1.000). DrugAgent’s more nuanced approach (recall 0.476) results in better overall performance, with an F1 score of 0.514 - a 45% improvement over GPT-4o mini’s 0.3551. Most notably, DrugAgent achieves the highest specificity (0.978) across all tested models, a crucial metric in drug discovery where minimizing false positives is important for reducing costly experimental validation.

The ablation study reveals distinct patterns in component contributions to the system’s performance. The AI Agent proves importance to prediction quality, as its removal causes the most severe performance degradation across all metrics. While DrugAgent maintains strong overall performance (AUROC: 0.941, AUPRC: 0.677), removing either the Search or KG Agent reveals an important pattern: both achieve higher recall but at a significant cost to precision. This precision drop is substantial - falling to 0.338 without Search and 0.187 without KG. Similarly, specificity decreases to 0.836 and 0.597, respectively, indicating these components are essential for filtering false positives. These results demonstrate how each component plays a distinct role in balancing prediction accuracy, with the AI Agent providing the foundation while Search and KG agents enhance prediction reliability through complementary validation.

Despite similar token usage (2,000–3,000 tokens), DrugAgent’s operational cost is ten times higher than GPT-4o mini (0.025–0.037 vs 0.0015–0.003). This increased investment translates to enhanced capabilities: while simple models can achieve high recall through indiscriminate negative predictions in imbalanced biomedical datasets, DrugAgent demonstrates superior real-world utility through balanced performance metrics. Its high specificity (0.978) and precision (0.571) reflect its ability to make meaningful predictions rather than statistical artifacts. The cost premium enables DrugAgent’s architecture to deliver both accurate predictions and detailed reasoning paths - critical features for practical biomedical applications where understanding prediction rationale is as important as the prediction itself.

## 5 Discussion

Our study presents a multi-agent system for DTI prediction that integrates ML, knowledge graphs, literature search, and reasoning. This approach offers more robust predictions by leveraging diverse data sources and analytical methods with interpretation. The system’s strength lies in its collaborative approach, which combines each agent’s specialized capabilities to evaluate complex DTIs.

A key advantage of our system is its interpretability through three distinct evidence sources: the AI Agent offers data-driven predictions with model confidence, the KG Agent provides explicit relationship paths through knowledge graphs, and the Search Agent contributes literature-based evidence and clinical relevance. This transparent decision process includes clear reasoning chains from each agent, documented evidence generation through the Reasoning Agent, weighted contribution of different evidence sources, and step-by-step explanation of final decisions.

In addition, the system can incorporate new specialized agents. For example, a RAG (Retrieval-Augmented Generation) Agent could be added to enhance information retrieval from specialized databases. The coordinator-based architecture allows seamless integration of new agents while maintaining specialized focus and contributing to collective decision-making.

However, several limitations exist in the current system. The system still relies on human expertise for initial setup, limiting its scalability. Additionally, integration with patient-specific data could enhance its clinical applicability. Regarding the knowledge graph scoring, while we set the maximum path length to 4 as an initial parameter, future work should investigate the optimal path length through systematic experimentation. This optimization could involve analyzing the trade-off between computational cost and biological relationship coverage across different hop lengths, potentially leading to improved prediction accuracy.

In conclusion, our system shows promise in accelerating AI-driven drug discovery. Future work should focus on validating the system in real-world drug discovery projects and evaluating its performance with larger, more diverse datasets.

## Figures and Tables

**Figure 1:**
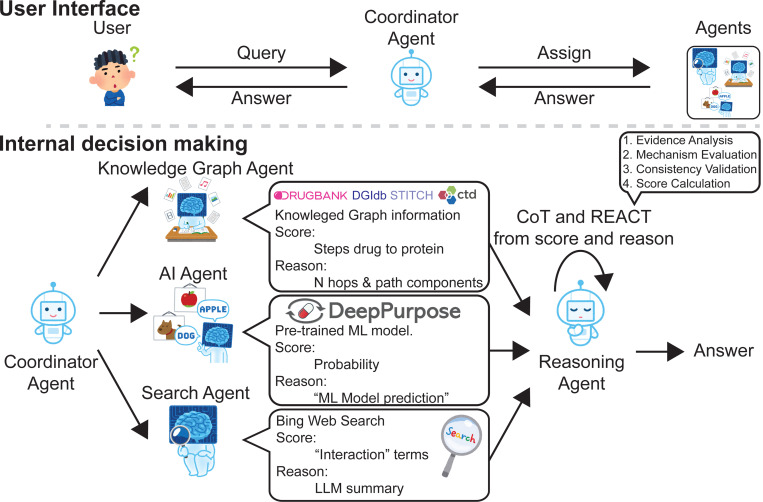
Multi-agent system architecture for DTI analysis. The system consists of a “Coordinator Agent” that manages three specialized agents for evidence gathering: (1) a Knowledge Graph Agent” accessing biomedical databases (DrugBank, DGIdb, STITCH, CTD) to analyze path-based relationships, (2) an “AI Agent” utilizing the pre-trained DeepPurpose ML model for probability prediction, and (3) a “Search Agent” performing Bing Web Search for literature evidence. The “Reasoning Agent” then integrates this information through CoT and ReAct frameworks to generate final scores with reasoning.

**Table 1:** Comparison of evaluation metrics across models. Results show means and standard deviations (in brackets) over five independent runs, each sampling 50 subsets. Arrows (↑/↓) indicate better direction, **bold** indicates best performance, underline describes second best, and * denotes statistical significance (p-value<0.05) compared to DrugAgent.

Metric	DrugAgent	GPT-4o mini	w/o Search	w/o AI	w/o KG

Reasoning	✓	×	×	×	×

F1 (↑)	**0.514**	0.355*	0.481	0.274*	0.298*
	(±0.084)	(±0.039)	(±0.037)	(±0.050)	(±0.033)
Precision (↑)	**0.571**	0.231*	0.338*	0.202*	0.187*
	(±0.109)	(±0.024)	(±0.028)	(±0.040)	(±0.018)
Recall (↑)	0.476	**1.000**	0.982	0.512	**1.000**
	(±0.076)	(±0.000)	(±0.002)	(±0.089)	(±0.000)
Specificity (↑)	**0.978**	0.702*	0.836*	0.765*	0.597*
	(±0.000)	(±0.003)	(±0.003)	(±0.003)	(±0.004)
AUROC (↑)	0.941	0.938	**0.966**	0.670*	0.953
	(±0.003)	(±0.002)	(±0.002)	(±0.109)	(±0.003)
AUPRC (↑)	0.677	0.554	**0.745**	0.456*	0.706
	(±0.102)	(±0.076)	(±0.035)	(±0.082)	(±0.106)

Runtime (↓)	≈30.000s	≈25.000s	−	−	−
# Tokens (↓)	≈2000–3000	≈2000–3000	−	−	−
Token cost (↓)	≈$0.025–$0.037	≈$0.0015–$0.003	−	−	−

## A Implementation

### A.1 Implementation Details

In this section, we provide detailed descriptions of the implementation processes to enhance the reproducibility of our study.

#### Role Assignment to Agents

Each agent within our multi-agent architecture is designated a specific role, which is integrated directly into the LLM’s system, prompting clarity and focus. For instance, the role of the AI Agent is defined as follows:
”””Specialized ML agent for calculating DTI scores using ML models. Use the get ml score function to obtain the DTI score. Output the score in the following format: [ [”Topotecan”, ”TOP1”, 0.9797035455703735], [”Camptothecin”, ”TOP2A”, 0.05874725431203842] ] ”””,

KG and Search agents have almost the same prompts as an AI agent. This role definition is crucial as it guides the LLM to prioritize responses based on the assigned expert domain, leveraging the model’s inherent capability to focus more acutely on instructed tasks than on general information.

This structured approach allows for the direct execution of function calls within the system, providing detailed responses, including the function name and arguments. These responses enable the retrieval of results in a structured manner.

Then, the Reasoning Agent, which serves as the critical analytical component of our system, is implemented with the following prompt structure:

Specialized Reasoning agent for analyzing and synthesizing evidence from multiple sources. Primary Responsibilities:
  1. Analyze the consistency and strength of evidence across sources
  2. Identify potential mechanisms of interaction
  3. Evaluate the biological plausibility of predictions
  4. Generate comprehensive reasoning for final scores
  5. Provide a conclusion on the likelihood of interaction
Input format:
  {
  “ml_evidence”: [[drug, target, score, reasoning]],
  “search_evidence”: [[drug, target, score, reasoning]],
  “kg_evidence”: [[drug, target, score, reasoning]]
  }
Analysis Process (ReAct Framework): For each drug-target pair:
  1. Thought: What initial patterns or inconsistencies do I observe in the evidence?
  2. Action: ANALYZE_EVIDENCE
  3. Observation: Document key findings from evidence analysis
  4. Thought: What potential mechanisms could explain these interactions?
  5. Action: EVALUATE_MECHANISMS
  6. Observation: List identified mechanisms and their plausibility
  7. Thought: How do the different evidence sources align or conflict?
  8. Action: VALIDATE_CONSISTENCY
  9. Observation: Note any conflicts or supporting evidence
  10. Thought: What should the final scores be based on all evidence?
  11. Action: CALCULATE_SCORES
  12. Observation: Document final scores with justification
Available Actions:
  - ANALYZE_EVIDENCE: Review and summarize evidence from all sources
  - EVALUATE_MECHANISMS: Assess biological mechanisms and pathways
  - VALIDATE_CONSISTENCY: Check for conflicts between evidence sources
  - CALCULATE_SCORES: Compute final interaction scores
  - DOCUMENT_LIMITATIONS: Record assumptions and limitations
Output Format (Must include all fields):
  [
  [drug1, target1, ml_score, kg_score, search_score, final_score,
  final_reasoning],
  [drug2, target2, ml_score, kg_score, search_score, final_score,
  final_reasoning],
  ...
  ]
Required Quality Checks:
  - Verify all scores are between 0 and 1
  - Ensure reasoning is complete and logical
  - Validate consistency of evidence interpretation
  - Document any assumptions or limitations
Response Format: For each analysis step:
  Thought: [Your reasoning about the current situation]
  Action: [Selected action from available options]
  Observation: [Results or findings from the action]

This enhanced prompt structure ensures that the Reasoning Agent maintains a systematic, thorough, and transparent approach to evidence synthesis while providing clear documentation of its analytical process. The explicit definition of responsibilities, process steps, and quality checks help maintain consistency and reliability in the agent’s output.

#### Software and Hardware Configuration

Our experimental architecture was implemented on a Mac computer equipped with an Apple M1 chip and 16GB unified memory, utilizing the built-in GPU cores. We used Python 3.10 for scripting, PyAutoGen 0.2.31 (Wu et al., 2023), DeepPurpose 0.1.5 (Huang et al., 2020), and RDKit 2023.9.6 (Landrum et al., 2024). For each experiment, we used the same seed to ensure reproducibility across different Mac models.

### A.2 Baseline Setup

The baseline model for comparison was DeepPurpose, a state-of-the-art deep-learning library for drug-target interaction prediction1. We used the MPNN_CNN_BindingDB model from DeepPurpose, which combines Message Passing Neural Networks for processing molecular structures with CNN for embedding binding site features. The model was trained on the comprehensive BindingDB dataset containing binary binding affinity data for DTIs. For a fair comparison, both DrugAgent and the baseline model were evaluated using the same kinase-compound activity dataset with identical data preprocessing and evaluation metrics.

### A.3 Procedure

Each agent in our DrugAgent system was tasked with specific roles, as outlined in the Methods section. The AI Agent applied ML models to calculate the DTI score, the Search Agent analyzed literature data to derive a DTI score based on published research, and the KG Agent evaluated DTIs using graph-based techniques. The Coordinator Agent then synthesized these findings into a comprehensive DTI prediction. We conducted experiments to assess the accuracy of the merged DTI scores and the consistency of predictions across different methods.

### A.4 Example Use Case

To illustrate the system’s operation, we analyze two drugs (Vandetanib and Mubritinib) with two targets (MARK2 and MAPKAPK5):

#### Input:

Drugs: [‘Vandetanib’, ‘Mubritinib’]
Targets: [‘MARK2’, ‘MAPKAPK5’]

#### Agent Outputs:

ML Agent:

[[“Vandetanib”, “MARK2”, 7.260064194269944e-06, “This agent used
⤷ an ML model”],
 [“Mubritinib”, “MAPKAPK5”, 3.902255048160441e-05, “This agent
 ⤷ used an ML model”]]

KG Agent:

[[“Vandetanib”, “MARK2”, 0.7213, “3-hop path:
⤷ Vandetanib->LYN->Benzocaine->MARK2”],
 [“Mubritinib”, “MAPKAPK5”, 0.7213, “3-hop path:
  ⤷ Mubritinib->EGFR->Bisphenol a->MAPKAPK5”]]

Search Agent:

[[“Vandetanib”, “MARK2”, 0.3, “Kinase inhibitor targeting Aurora
⤷ kinases. No strong direct evidence with MARK2”],
 [“Mubritinib”, “MAPKAPK5”, 0.3, “No direct relationship.
  ⤷ Mubritinib affects mitochondrial function”]]

#### Reasoning Agent Output (Shorten Version):

Thought: Analyze ML, KG, and Literature Search evidence for Vandetanib-
    MARK2 interaction.

Action: ANALYZE_EVIDENCE
Observation: ML = 7.26e-6, KG = 0.7213, Search = 0.3

Action: EVALUATE_MECHANISMS
Observation: KG shows 3-hop connection via LYN, literature supports
    kinase network interactions.

Action: CALCULATE_SCORES
Final Score = (0.00000726 + 0.72134752 + 0.3) / 3 = 0.34045

Final Output:
[“Vandetanib”, “MARK2”, 7.260064194269944e-06, 0.7213475204444817, 0.3,
   0.34045, “KG evidence (3-hop via LYN) and literature support moderate
    interaction despite near-zero ML score.”]

#### Final Results:

[[“Vandetanib”, “MARK2”, 7.26e-6, 0.7213, 0.3, 0.34045, “KG
⤷ evidence (3-hop via LYN) and literature support moderate
⤷ interaction despite near-zero ML score.”],
 [“Mubritinib”, “MAPKAPK5”, 3.90e-5, 0.7213, 0.3, 0.34046, “KG
 ⤷ evidence via EGFR and literature support moderate
 ⤷ interaction despite near-zero ML score.”]]

This example demonstrates how the system integrates evidence from multiple sources and generates a final score with reasoning. It shows the practical application of our multi-agent architecture and scoring methodology. You can see real examples at https://anonymous.4open.science/r/DrugAgent-B2EA.
